# Post-Traumatic Stress Disorder in Scabies Patients: The Overlooked Part of a Global Outbreak

**DOI:** 10.7759/cureus.78063

**Published:** 2025-01-27

**Authors:** Huriye Aybüke Koç, Yesim Olgun

**Affiliations:** 1 Dermatology, Prof. Dr. A. Ilhan Ozdemir State Hospital, Giresun, TUR; 2 Psychiatry, Prof. Dr. A. Ilhan Ozdemir State Hospital, Giresun, TUR

**Keywords:** dermatology life quality index, outbreak, post-traumatic stress disorder, psychodermatology, scabies

## Abstract

Scabies is a parasitosis caused by *Sarcoptes scabiei* var *hominis. *The most important clinical symptom of scabies is severe itching, which can cause insomnia, lack of concentration, and disruption in social life in patients. In addition to exposure to a traumatic stressor, post-traumatic stress disorder (PTSD) can also occur with various diseases that impair quality of life. With this study, we aimed to investigate whether scabies cause PTSD.

The Dermatology Life Quality Index (DLQI) and Hospital Anxiety Depression Scale (HADS) were applied to the scabies patients at the time of initial diagnosis. The Impact of Events Scale-Revised (IES-R), HADS, Peritraumatic Dissociation Scale (PDEQ), and Life Events Checklist (LEC) were administered to the patients who came for control one month later.

Among the 82 research participants, 53 (64.7%) patients had moderately to severely impacted quality of life, with a mean DLQI total score of 8.57±5.29. Also, 53.7% of patients diagnosed with scabies had a significantly high IES-R score for PTSD. In the group with PTSD (+), DLQI, anxiety and depression scores, PDEQ score, and LEC score were higher both at the time of diagnosis and one month after diagnosis.

In our cross-sectional study, a significantly higher IES-R score was found in patients with scabies in terms of PTSD. We believe that scabies patients should be examined prospectively in terms of psychiatric morbidities and dermatologists' awareness should be increased in this sense.

## Introduction

Scabies is a parasitosis caused by *Sarcoptes scabiei *var *hominis*. Itchy skin lesions appear as a result of either direct or indirect transfer [1-3]. The most important clinical symptom of scabies is severe itching, and it may cause conditions such as insomnia, decreased concentration, and social stigma in patients [4,5].

Typically, exposure to a traumatic stressor, such as an actual or imminent death, a serious injury, or sexual abuse, causes post-traumatic stress disorder (PTSD) [6]. Among dermatological diseases, there are studies examining the relationship between various diseases, such as psoriasis, vitiligo, urticaria, recurrent herpes simplex infection, pruritus, and alopecia areata, and PTSD [7-11].

According to studies, PTSD can be linked to non-traumatic events such as declining health-related quality of life, psychological distress brought on by social stigma, financial hardship, relapse, illness exacerbation, and adverse drug reactions, which may hasten the onset of PTSD. Again, in these studies, it was found that sleep disorders significantly impair the quality of life and pose a risk for PTSD.

Sleep disturbances seen in patients diagnosed with scabies, social stigma, resistance to treatments, and increased re-infections due to the inability to prevent infection in the fight against the epidemic suggest that these may be risk factors for PTSD in patients. Studies have shown that scabies affects the quality of life of patients and may trigger depression and anxiety [12,13]. However, to our knowledge, the relationship between scabies and PTSD has never been investigated before. Therefore, it was aimed to investigate the relationship with PTSD in the one-month follow-up of patients diagnosed with scabies.

## Materials and methods

Study design

The study included 82 patients with scabies who visited the dermatology outpatient clinic at Prof. Dr. Ilhan Ozdemir State Hospital, Giresun, Turkey, between June 1 and October 15, 2023. Following their disclosure of study details, the patients completed permission papers attesting to their voluntary participation in the trial. Approval of Giresun Training and Research Hospital Clinical Research Ethics Committee was received for the study (13.03.2023/12).

Patients

**Figure 1:**
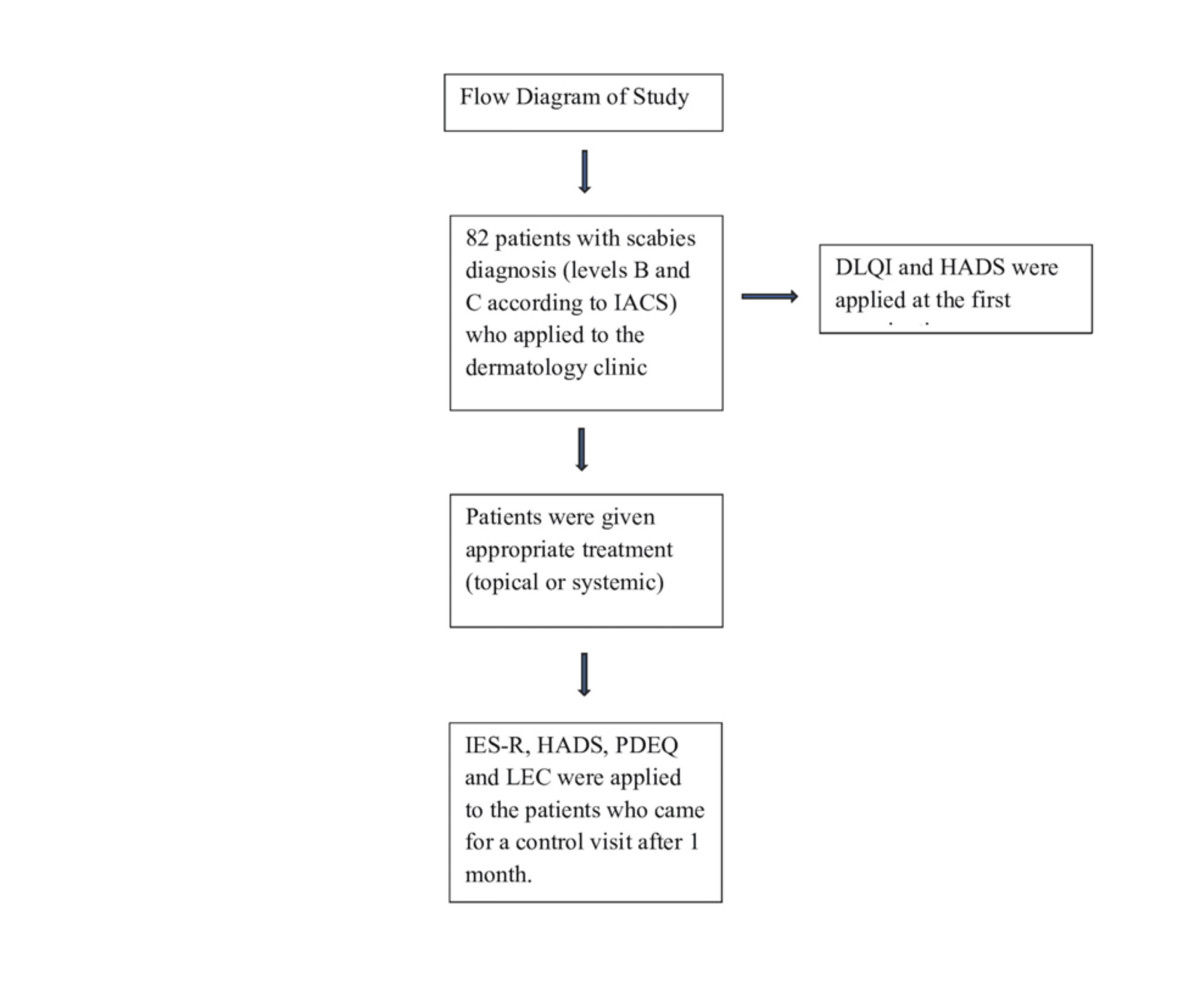
Flow diagram of the study DLQI, Dermatology Life Quality Index; HADS, Hospital Anxiety Depression Scale; IACS, IES-R, Impact of Events Scale-Revised; LEC, Life Events Checklist; PDEQ, Peritraumatic Dissociation Scale

The research included patients diagnosed with scabies who were older than 18 years. Those under the age of 18 years and those with comorbid neurological and/or systemic disease were not included in the study. The International Alliance for the Control of Scabies (IACS) 2020 consensus criteria were used to identify patients who applied to the dermatology outpatient clinic and were diagnosed with clinical (level B) and suspected (level C) scabies [14]. Findings were recorded in the sociodemographic data form. After the first examination, the Dermatology Life Quality Index (DLQI) and Hospital Anxiety Depression Scale (HADS) were applied to the patients (Figure 1). As a treatment for scabies, sulfur mixture to be applied for three consecutive days and/or two courses of ivermectin tablets at 10-day intervals was preferred. Patients were noted as those who had never received scabies treatment before and those who treated scabies themselves because they had scabies before or without consulting a doctor. At the follow-up visits after one month, the Impact of Events Scale-Revised (IES-R), HADS, Peritraumatic Dissociative Experience Questionnaire (PDEQ), and Life Events Checklist (LEC) were administered to the patients. A dermatologist and a skilled psychiatrist assessed the anklet results, and all questionnaires were administered to the patients under the supervision of a physician.

Dermatology Life Quality Index (DLQI)

The DLQI consists of 10 elements that are focused on six domains: therapy, symptoms and feelings, personal connections, work and school, leisure, and everyday activities. The total score ranges from 0 to 30 on a 4-point Likert scale (0, "not at all," 1, "somewhat," 2, "a lot," and 3, "very much"). Increasing scores mean deterioration in quality of life. Furthermore, each domain was deemed to have impairment if the score was 1 or higher. A validity study of the DLQI in Turkey was carried out [15].

Hospital Anxiety Depression Scale (HADS)

The HADS [16] is a scale used for screening purposes. The 14-item scale includes the Anxiety subscale and the Depression subscale, which consist of mixed seven items each. There is a four-point on the scale. According to the information provided, each subscale has a normal range of 0 to 7 points, an indication of anxiety or depression present from 8-10 points, and a possibility of anxiety or depression from 11 points and above [17]. HADS has previously been validated for the Turkish population [18].

Impact of Events Scale-Revised (IES-R)

A 22-item self-report questionnaire called the IES-R evaluates hyperarousal, avoidance, and intrusive symptoms and yields a total score for subjective distress associated with a traumatic event. The scale, consisting of 22 items, has a scoring system between 0 and 4 (0 = not at all, 4 = very much). An increase in the total score obtained from the scale means that the patient experiences more PTSD symptoms. According to studies, scores of 33 and above on the scale indicate a diagnosis of PTSD and scores of 24 and above indicate clinically significant findings [19]. An investigation was carried out on the Turkish version's validity and trustworthiness [20]. We made slight adjustments to the Turkish version of the IES-R to cater to the nature of the event under investigation (where applicable, substituting the word "scabies" for "event"). Those with a cut-off point of 33 and above were considered to have PTSD (+), and the patients were divided into two groups PTSD (+) and PTSD (-).

Peritraumatic Dissociative Experience Questionnaire (PDEQ)

The PDEQ was developed to determine the dissociative symptoms experienced by a person during and immediately after a traumatic event. There are 10 items altogether on the scale. High scores on the scale indicate increased dissociation during the traumatic event. An investigation was carried out on the Turkish version's authenticity and trustworthiness. We made a minor modification in the PDEQ to say “We aim to learn about your feelings about scabies” [21].

Life Event Checklist (LEC)

A 17-item self-report test called the LEC evaluates exposure to 16 events that have been linked to PTSD as well as one other extremely stressful event that is not on the list [22]. The number of the following items (i.e., "personally experienced," "witnessed," "learned," and "as part of my job") that each subject checked determined their score for past traumatic experiences.

Statistical analysis

The statistical analyses were carried out using SPSS 26.0 for Windows (IBM Corp., Armonk, NY, USA). Using analytical techniques (Kolmogorov-Smirnov/Shapiro-Wilk test) and visual aids (histogram and probability graphs), the appropriateness of the variables for a normal distribution was assessed. When a data set did not exhibit a normal distribution, the Mann-Whitney U test was utilized. A p-value of <0.05 was used as the statistical significance threshold.

## Results

The study included 82 patients diagnosed with scabies, of whom 46 (56.1%) were female and 36 (43.9%) were male. The average age was 32.8±14.53. Table 1 shows the further sociodemographic details about the patients. Patients with PTSD (+) and patients with PTSD (-) did not differ in terms of sociodemographic data, scabies diagnosis, having previously been diagnosed with scabies and receiving treatment, treatments given for scabies, or a history of psychiatric illness. Compared to scabies patients with PTSD (-), only the percentage of family history of psychiatric disease was significantly higher in scabies patients with PTSD (+) (p=0.01) (Table 1).

**Table 1:** Sociodemographic characteristics of the patients included in the study PSTD, post-traumatic stress disorder; χ^2^, chi-square test *According to the International Alliance for the Control of Scabies (IACS) 2020 consensus criteria, patients with clinical diagnosis of scabies were evaluated as B and patients with suspected scabies were evaluated as C.

	PTSD (-) (n)	PTSD (+) (n)	Total (n)	p-Value	χ^2^
Sex and habits					
Female	18	28	46	0.139	2.191
Male	20	16	36		
Smoking	18	15	33	0.319	0.994
Alcohol	6	8	14	1	0
Marital status					
Married	21	20	41	0.647	0.871
Single	17	24	41		
Occupation					
Working	16	12	28	0.368	2.002
Nonworking	14	20	34		
Student	8	12	20		
Scabies diagnosis					
B*	22	32	54	0.158	1.995
C*	16	12	28		
Previous history of scabies					
Yes	4	7	11	0.476	0.509
No	34	37	71		
Previous history of scabies treatment					
Yes	7	10	17	0.631	0.230
No	31	34	65		
Treatment of choice					
Only topical	33	33	66	0.177	1.821
Topical and systemic	5	11	16		
Previous psychiatric disorder history					
Present	10	15	25	0.446	0.582
Absent	28	29	57		
Psychiatric disorder history in the family					
Present	0	7	7	0.01	6.610
Absent	38	37	75		

The average DLQI total score was 8.57±5.29. While the quality of life of 29 (35.3%) patients was slightly or not affected at all, 53 (64.7%) patients were moderately to severely affected. The average score was 6.21±5.27 in patients with PTSD (-) and 10.61±5.31 in patients with PTSD (+). A statistically significant difference was found in DLQI between those with PTSD (-) and those with PTSD (+) (p<0.001). Among those with PTSD (-) and those with PTSD (+), the most deterioration in DLQI scores was in the symptoms and emotions (questions 1-2) domain. In patients with PTSD (+), after symptoms and emotions, there was deterioration in daily activities, work and school, and personal relationships, while in patients with PTSD (-) there was deterioration in work and school, daily activities, and personal relationships (Table 2).

**Table 2:** Scores of each DLQI domain in patients with PTSD (+) and PTSD (-) PSTD, post-traumatic stress disorder; DLQI, Dermatology Life Quality Index; Q, questions

	PTSD (-)	PTSD (+)	p-Value
DLQI	6.21±5.27	10.61±5.31	<0.001
Symptoms and emotions (Q 1-2)	2.21±1.51	3.09±1.29	0.002
Daily activities (Q 3-4)	0.95±1.18	2.45±1.38	0.001
Free time (Q 5-6)	0.63±1.14	1.36±1.33	0.003
Work and school (Q 7)	1.05±1.13	1.86±1.02	0.001
Personal relationships (Q 8-9)	0.92±1.21	1.43±1.40	0.038
Treatment (Q 10)	0.47±0.64	0.41±0.62	0.622

The median HADS anxiety score at the time of initial diagnosis was 6.11±3.43 in patients with PTSD (-), while it was 8.32±3.41 in patients with PTSD (+). At the time of initial diagnosis, the median HADS depression score in patients with PTSD (-) was 5.11±2.93, while it was 6.66 ±3.11 in patients with PTSD (+). A statistically significant difference was found in HADS anxiety and depression scores at the time of initial diagnosis between those with PTSD (-) and those with PTSD (+) (p=0.011 and p=0.031, respectively) (Table 3). One month after diagnosis, the median HADS anxiety score in patients with PTSD (-) was 4.53±4.24, while it was 8.64±3.74 in patients with PTSD (+). One month after diagnosis, the median HADS depression scores in patients with PTSD (-) was 3.82 ± 3.47, while it was 7.80 ± 3.05 in patients with PTSD (+). A statistically significant difference was found in HADS anxiety and depression scores between those with PTSD (-) and those with PTSD (+) one month after diagnosis (p<0.001) (Table 3). The average PDEQ score was 13.50±3.65 in patients with PTSD (-), while it was 16.84±5.29 in patients with PTSD (+). The PDEQ scores of those with PTSD (-) and those with PTSD (+) differed statistically significantly (p=0.02) (Table 3). The average LEC score was 3.79±4.13 in patients with PTSD (-), while it was 7.30±6.14 in patients with PTSD (+). The PDEQ scores of those with PTSD (-) and those with PTSD (+) differed statistically significantly (p=0.02) (Table 3).

**Table 3:** DLQI, HADS, PDEQ, and LEC scale scores of patients with PTSD (+) and PTSD (-) PTSD, post-traumatic stress disorder, HADS, Hospital Anxiety Depression Scale, PDEQ, Peritraumatic Dissociative Experience Questionnaire, LEC, Life Event Checklist

	PTSD (-)	PTSD (+)	p-Value
HADS (first diagnosis)	11.29±5.72	15.00±5.53	0.016
Anxiety	6.11±3.43	8.32±3.41	0.011
Depression	5.11±2.93	6.66±3.11	0.031
HADS (after one month)	8.34±6.87	16.39±5.60	<0.001
Anxiety	4.53±4.24	8.64±3.74	<0.001
Depression	3.82±3.47	7.80±3.05	<0.001
PDEQ	13.50±3.65	16.84±5.29	0.02
LEC	3.79±4.13	7.30±6.14	0.02

A negative and significant relationship was found between those who had not received scabies treatment before and the DLQI total score, symptoms and emotions (questions 1-2), personal relationships (questions 8-9), and treatment (question 10) variables (rs=-0.222/p=0.045, rs=-0.268/p=0.015, rs=-0.293/p=0.008, rs=-0.324/p=0.003 respectively). Accordingly, lower total DLQI score, DLQI scores 1-2, DLQI scores 8-9, and DLQI score 10 were observed in those who had not received scabies treatment before. A negative and significant relationship was found between those with a previous family history of psychiatric illness and personal relationships (questions 8-9) variables (rs=--0.300/p=0.006). Accordingly, lower DLQI 8-9 scores were observed in those with a family history of psychiatric disease (Table 4).

**Table 4:** Spearman rank correlation coefficients (rs) for DLQI scores DLQI, Dermatology Life Quality Index

	DLQI		DLQI 1-2		DLQI 3-4		DLQI 5-6		DLQI 7		DLQI 8-9		DLQI 10	
	r_s_	p	r_s_	p	r_s_	p	r_s_	p	r_s_	p	r_s_	p	r_s_	p
Age	-0.007	0.947	-0.012	0.915	0.047	0.673	-0.030	0.792	-0.002	0.983	-0.040	0.722	-0.030	0.788
Sex	0.059	0.596	0.049	0.660	0.142	0.202	-0.012	0.917	0.032	0.773	0.001	0.999	-0.045	0.686
Education level	0.188	0.090	0.134	0.230	0.020	0.857	0.131	0.240	0.187	0.092	0.159	0.154	0.213	0.055
Marital status	0.133	0.232	0.130	0.245	0.064	0.566	0.042	0.706	0.077	0.492	0.126	0.258	0.106	0.343
Occupation	0.47	0.678	0.020	0.858	-0.017	0.883	0.024	0.833	0.161	0.149	0.069	0.541	0.130	0.245
Previous psychiatric disorder diagnosis	0.001	0.996	0.001	0.99	-0.065	0.559	-0.002	0.983	0.012	0.913	-0.068	0.546	-0.016	0.888
Psychiatric disorder history in the family	-0.209	0.590	-0.118	0.292	-0.200	0.072	-0.198	0.075	-0.134	0.231	-0.300	0.006	0.172	0.123
Scabies diagnosis	0.077	0.492	0.056	0.617	0.100	0.370	0.168	0.132	-0.021	0.853	- 0.037	0.740	0.172	0.123
Previous scabies history	0.071	0.529	-0.076	0.499	0.047	0.673	0.039	0.728	-0.082	0.462	-0.182	0.102	-0.180	0.106
Previous scabies treatment history	-0.222	0.045	-0.268	0.015	-0.085	0.449	-0.062	0.580	-0.123	0.273	-0.293	0.008	-0.324	0.003
Scabies treatment	0.024	0.829	0.001	0.991	-0.073	0.516	0.092	0.411	0.034	0.759	0.056	0.617	-0.18	0.870

When PTSD (+) patients' anxiety and depression scores were compared one month after treatment, there was no statistically significant difference in terms of age, marital status, education level, occupation, smoking/alcohol habit, scabies diagnosis, prior scabies diagnosis and treatment, scabies treatments received, prior history of psychiatric illness, and family history of psychiatric disease (Table 5).

**Table 5:** Comparison of anxiety and depression scores one month after treatment in the group with PTSD (+) according to sociodemographic characteristics PTSD, post-traumatic stress disorder *According to the International Alliance for the Control of Scabies (IACS) 2020 consensus criteria, patients with clinical diagnosis of scabies were evaluated as B and patients with suspected scabies were evaluated as C.

	Anxiety one month after treatment		Depression one month after treatment	
	r_s_	p-Value	r_s_	p-Value
Age	0.226	0.141	0.182	0.237
Sex	-0.060	0.700	-0.171	0.267
Education level	-0.046	0.765	-0.199	0.196
Marital status	-0.050	0.747	-0.276	0.069
Occupation	-0.146	0.344	-0.154	0.317
Previous psychiatric disorder diagnosis	-0.275	0.071	0.069	0.658
Psychiatric disorder history in the family	-0.030	0.849	0.227	0.138
Scabies diagnosis (B/C)*	0.206	0.180	0.162	0.292
Previous scabies history	0.017	0.912	-0.111	0.472
Previous scabies treatment history	-0.131	0.397	-0.196	0.202
Scabies treatment (topical/systemic)	-0.222	0.147	0.186	0.227

## Discussion

To the best of our knowledge, this is the first research article examining PTSD in scabies patients. Prior research has demonstrated that scabies sufferers experience anxiety and sadness in addition to a decreased quality of life [5,12,22,23]. In our study, the average DLQI total score was 8.57±5.70, and the patients' quality of life was moderately affected. Previous studies have shown that the quality of life of patients with scabies is moderately impaired, consistent with our results [5,12,22]. In the study conducted by Paudel et al., the mean DLQI score was reported as 12.91, which was higher than our result [23]. This elevation was thought to be related to secondary bacterial infections occurring in the patient's lesions caused by scabies in this study. In DLQI scores, the most deterioration occurred in the symptoms and emotions (questions 1-2) domain. In previous studies on the quality of life of scabies, it was observed that the most deterioration was in the area of symptoms and emotions, similar to our results [5,12,24].

There was no discernible correlation between the DLQI total score and sociodemographic data, diagnosis of scabies, previous history of scabies, treatments given for scabies, previous psychiatric illness, and a family history of psychiatric disease. A negative and significant relationship was found only between those who had not received any previous scabies treatment and the total DLQI score (rs=-0.222, p=0.045). Accordingly, lower DLQI scores were observed in those who had not received scabies treatment before. In addition, in our study, lower scores were observed in the areas of symptoms and emotions (questions 1-2), personal relationships (questions 8-9), and treatment (question 10) in those who had not received scabies treatment before (rs=-0.268/p=0.015, rs=-0.293/p=0.008, rs=-0.324/p=0.003, respectively). Patients who had not been treated for scabies before consisted of patients whose scabies disease had just started and who were admitted to the hospital for the first time. The group that had previously received scabies treatment consisted of patients who had previously received scabies treatment under the supervision of a doctor or on their own and did not respond to treatment, and patients who were infected with scabies again even if they benefited from the treatment. The course of the disease was much shorter in the group that had not previously received scabies treatment than in the group that had previously received scabies treatment, was unresponsive to treatment, or was re-infected with scabies. They experienced the difficulty of applying topical treatments for scabies only once and were less exposed to the difficulty of treatment compared to the group who had previously received scabies treatment. Previous research has demonstrated that prolonged illness and repeated treatments have a detrimental impact on quality of life, which is consistent with our findings [12,22].

There were no differences between patients with and without disorders in each domain of DLQI in terms of gender, age, marital status, education level, occupation, smoking and alcohol habits, diagnosis of scabies, previous history of scabies, treatments given for scabies, and history of previous psychiatric illness. We came across studies in the literature that did not find differences similar to our study in terms of sociodemographic data [5,12] and found differences in terms of gender [22,24]. All these studies were conducted in different geographies and ethnic groups, and we thought that the different results might be related to this.

In patients with a family history of mental illness, a negative and significant link was also discovered between the DLQI's personal relationship (items 8-9) domain (rs=-0.300, p=0.006). Accordingly, those with a family history of psychiatric disease had lower scores in the field of personal relationships. We could not find any studies in this field in the literature. However, in a previous study [25], it was mentioned that psychiatric diseases cause a decrease in social relations. We assumed that these people, who have a family history of psychiatric illness and grew up with these parents, may already be deficient in social relationships, regardless of having scabies, and therefore there may not be a change in their lives in terms of personal relationships.

Among the patients participating in the study, a statistically significant increase in anxiety and depression scores was found in those with PTSD (+) compared to those with PTSD (-), both at the time of diagnosis and one month after the diagnosis. In previous studies investigating anxiety and depression in scabies patients, it was found that there was a significant increase in the anxiety and depression scores of scabies patients, consistent with the deterioration in quality of life. It is thought that patients' preference to remain socially isolated during the disease process contributes to this [12,13]. In our study, the average anxiety scores were above 8, especially in the group with PTSD (+), and it was determined that the patients were prone to anxiety.

Numerous dermatological conditions, including psoriasis, vitiligo, urticaria, recurrent herpes simplex infection, pruritus, alopecia areata, and PTSD, have been studied in the literature [7-11]. However, to our knowledge, there has been no previous study on PTSD in patients with scabies. In our study, more than half of the patients diagnosed with scabies (53.7%) had a significantly high IES-R score for PTSD.

DLQI scores, anxiety and depression scores, PDEQ scores, and LEC scores both at the time of diagnosis and one month after diagnosis were higher in patients with PTSD (p<0.001, p=0.011; p=0.031, p<0.001; p<0.001, p=0.02; p=0.02, respectively). It has been shown in many different studies that high PDEQ and LEC scores are positively associated with PTSD [26,27]. Similarly, in our study, both PDEQ and LEC scores of patients with scores of 33 and above on the IES-R scale were statistically significantly higher.

These findings suggested that, like other dermatological diseases, scabies negatively affects the quality of life and may cause psychiatric morbidities in the first few months after infection, in addition to causing epidemics as an infectious disease by predisposing patients to anxiety. This was consistent with other studies showing an increase in psychiatric morbidities after major epidemics [28,29]. This may be due to the fact that the anxiety and depression, social stigma, and deterioration in quality of life experienced by patients are precursors to serious psychiatric morbidities.

In terms of sociodemographic data, scabies diagnosis, prior scabies treatment history, scabies treatments received, and history of prior mental illness, there was no discernible difference between patients with PTSD (+) and patients with PTSD (-). Only the percentage of family history of psychiatric illness was significantly higher (p=0.01). This finding was consistent with studies showing that a previous history of psychiatric illness in the family predisposes to PTSD [26,30].

Among the study's limitations were the small patient population and the absence of a control group. Additionally, since it was an observational questionnaire-based cohort study, it limited our inference of causality in the relationship between scabies and PTSD. Again, our study did not include a large number of patients to assess differences in treatment that could have influenced the study results. Instead of using clinical interviews, we relied on patients' self-reports for our findings. To prove a conclusive link between scabies and PTSD, more prospective, thorough, and multicenter studies are required.

## Conclusions

Traditionally, PTSD was thought to result from trauma-related stressors. However, many recent studies have shown that PTSD can be associated with non-traumatic events. PTSD has not been investigated in scabies patients before, but in our study, we observed PTSD in more than half of the scabies patients with the results of the IES-R scale scores. In addition to previous studies showing that it affects the quality of life and predisposes patients to anxiety and depression, the assumption that it may cause PTSD suggests that patients with scabies should be examined prospectively in terms of psychiatric morbidities. It may be beneficial for dermatologists to increase their awareness of future psychiatric morbidities in patients with scabies and to offer early interventions in this context. Based on our study's findings, we think that, in order to prevent and lessen PTSD in scabies patients, it would be helpful to better explain the process to patients (explain that even if the treatment is fully applied, the itching will gradually decrease over time, etc.) and to produce educational videos about scabies produced by associations and individual physicians.
